# Prognostic value of disseminated tumor cells in unresectable pancreatic ductal adenocarcinoma: a prospective observational study

**DOI:** 10.1186/s12885-022-09714-x

**Published:** 2022-06-03

**Authors:** Oddmund Nordgård, Morten Lapin, Kjersti Tjensvoll, Satu Oltedal, Karin Hestnes Edland, Nicolay Bore Neverdahl, Dmitrij Fostenes, Herish Garresori, Nils Glenjen, Rune Smaaland, Bjørnar Gilje

**Affiliations:** 1 Department of Hematology and Oncology, Stavanger University Hospital, Stavanger, Norway; 2 Department of Chemistry, Bioscience and Environmental Technology, Faculty of Science and Technology, University of Stavanger, Stavanger, Norway; 3 Department of Oncology, Haukeland University Hospital, Bergen, Norway; 4 Present Address: Mosaic Oncology AS, Sandnes, Norway

**Keywords:** Disseminated tumor cells, DTC, Bone marrow, Survival, Prognosis, Pancreatic ductal adenocarcinoma, PDAC

## Abstract

**Background:**

Although pancreatic ductal adenocarcinoma (PDAC) rarely metastasizes to the skeleton, disseminated tumor cells have been detected in bone marrow samples from patients with this disease. The prognostic value of such findings is currently unclear. Thus, the current study aimed to clarify the prognostic information associated with disseminated tumor cell detection in samples from patients with PDAC.

**Methods:**

Bone marrow aspirates were obtained from 48 patients with locally advanced (*n* = 11) or metastatic (*n* = 37) PDAC, before and after 2 months of chemotherapy. Disseminated tumor cells were detected with an mRNA panel and quantitative reverse transcription PCR. We used the highest levels measured in healthy bone marrow (*n* = 30) as a threshold to define the positive detection of disseminated tumor cells. Progression-free and overall survival were analyzed with Kaplan–Meier and Cox proportional hazards regression analyses.

**Results:**

Disseminated tumor cells were detected in 15/48 (31%) bone marrow samples obtained before starting chemotherapy and in 8/25 (32%) samples obtained during chemotherapy. Patients with disseminated tumor cells detected before therapy had significantly shorter progression-free (*p* = 0.03; HR = 2.0) and overall survival (*p* = 0.03; HR = 2.0), compared to those without disseminated tumor cells in the bone marrow. When restricting disseminated tumor cell detection to keratins KRT7 and KRT8, the prognostic information was substantially stronger (*p* = 1 × 10^–6^; HR = 22, and *p* = 2 × 10^–5^; HR = 7.7, respectively). The multivariable Cox regression analysis demonstrated that disseminated tumor cell detection prior to treatment had independent prognostic value. In contrast, disseminated tumor cells detected during treatment did not have prognostic value.

**Conclusions:**

Disseminated tumor cells detected before commencing chemotherapy had prognostic value in patients with inoperable PDAC.

**Supplementary Information:**

The online version contains supplementary material available at 10.1186/s12885-022-09714-x.

## Background

Pancreatic ductal adenocarcinoma (PDAC) is a fatal malignancy; it is currently the seventh leading cause of cancer-related deaths worldwide [1]. The high lethality of the disease is related to its late detection; over 80% of cases are diagnosed at an incurable stage [2]. Although recent multidrug treatments have significantly improved survival for patients with metastatic PDAC, the 5-year survival rate remains less than 5% [2, 3]. Hence, there is a striking need for enhanced diagnostics and treatment alternatives for this patient group.

PDAC metastasizes primarily to the liver, peritoneum, and lungs. Less than 1% of patients experience bone metastases [4, 5]. Nevertheless, there is evidence that disseminated tumor cells (DTCs) can be found in the bone marrow of patients with PDAC (reviewed in [6]). DTCs have primarily been detected with immunocytochemical methods that utilize various antibodies against keratin proteins [6–8]. However, in a few studies, reverse transcription PCR has also been used to detect DTCs indirectly, using keratin mRNAs as DTC markers [9–11]. Although some studies have demonstrated that DTC detection had prognostic value in PDAC, the results have been conflicting [6, 8]. The discrepancies may be related to the choice of DTC enrichment and detection methods, the choice of DTC markers and other methodological differences.

To clarify the prognostic value of DTCs in PDAC, in the present study, we used a sensitive method for detecting DTCs in bone marrow samples from patients with locally advanced or metastatic PDAC. This method involved multi-marker, reverse transcription, quantitative PCR (RT-qPCR) with pre-amplification. We investigated the prognostic value of DTCs detected both before and during chemotherapy, and the efficacy of using various DTC markers in this regard.

## Methods

### Patients and samples

We prospectively recruited 48 patients that had been treated for locally advanced (*n* = 11) or metastatic (*n* = 37) histologically confirmed PDAC at Stavanger University Hospital (*n* = 42) or Haukeland (Bergen) University Hospital (*n* = 6), between September 2012 and December 2020. The clinicopathological characteristics of the patients are shown in Table 1. Written informed consent was obtained from all participants, and the project was approved by the Regional Committee for Medical and Health Research Ethics (2011/475).

**Table 1:** Baseline patient characteristics stratified according to pre-treatment DTC status

**Variable**	**No. of patients [*n* = 48]**	**DTC positive [*n* = 15]**	**DTC negative [*n* = 33]**	***P* value**
**Median age (range)**	67 [41–79]	63 [46–74]	67 [41–79]	0.5
**Sex (%)**				0.5
Female	20 (42)	5 (33)	15 (45)	
Male	28 (58)	10 (67)	18 (55)	
**Primary tumor location (%)**				0.8
Head	19 (40)	4 (27)	15 (45)	
Body	7 (15)	3 (20)	4 (12)	
Tail	7 (15)	2 (13)	5 (15)	
Unknown^a^	15 (31)	6 (40)	9 (27)	
**Clinical T stage (%)**				**0.03**
T1	1 (2)	0 (0)	1 (3)	
T2	14 (29)	8 (53)	6 (18)	
T3	7 (15)	3 (20)	4 (12)	
T4	20 (42)	3 (20)	17 (52)	
Tx^a^	6 (13)	1 (7)	5 (15)	
**Clinical N stage (%)**				0.2
N0	19 (40)	7 (47)	12 (36)	
N1	16 (33)	4 (27)	12 (26)	
N2	3 (6)	0 (0)	3 (9)	
Nx	10 (21)	4 (27)	6 (18)	
**Clinical M stage (%)**				0.5
M0	11 (23)	2 (13)	9 (27)	
M1	37 (77)	13 (87)	24 (73)	
**Metastasis location (%)**				0.5
Only liver	24 (50)	9 (60)	15 (45)	
Only lung	3 (6)	2 (13)	1 (3)	
Multiple organs^b^	3 (6)	1 (7)	2 (6)	
Peritoneal carcinomatosis	7 (15)	1 (7)	6 (18)	
None	11 (23)	2 (13)	9 (27)	
**ECOG status (%)**				0.5
0	13 (27)	3 (20)	10 (30)	
1	29 (60)	10 (67)	19 (58)	
2	6 (13)	2 (13)	4 (12)	
**First-line treatment (%)**^**c**^				0.3
Gemcitabine	5 (10)	4 (27)	1 (3)	
Gemcitabine + Abraxane	24 (50)	7 (47)	17 (52)	
FOLFIRINOX	19 (40)	4 (27)	15 (45)	
**Second-line treatment (%)**				0.4
Yes	18 (38)	4 (27)	14 (42)	
No	30 (62)	11 (73)	19 (58)	
**Study site (%)**				0.4
Stavanger	42 (88)	12 (80)	30 (91)	
Bergen	6 (13)	3 (20)	3 (9)	

^a^Categories unknown and similar were not included in statistical testing

^b^Not including carcinomatosis

^c^Testing FOLFIRINOX combination versus other treatments

All patients were treated with chemotherapy according to Norwegian national guidelines [12]. Eighteen patients also received second-line treatments, after progression occurred on the first-line treatment. Treatment responses was monitored with radiological imaging, based on the RECIST 1.1 criteria [13].

For the present study, we collected patient follow-up data from medical records. Information on the time of death was also obtained from the hospital records, which were frequently updated, based on information from the National Registry in Norway. The last follow-up was recorded in June 2021, and the median follow-up time was 7.0 months (range 0.30–64 months).

Bone marrow samples (9 ml in EDTA tubes) were drawn unilaterally, from the posterior iliac crest under local anesthesia, before starting chemotherapy (*n* = 48) and after eight weeks of treatment (*n* = 25). In addition, bone marrow aspirates were obtained from 30 partially age-matched healthy individuals, which were included as a control group. The control group were self-reported free for any malignancy, had median age 54 years (range 22–69 years), and consisted of 20 women and 10 men, that signed a written informed concent.

Mononuclear cells were enriched from the bone marrow samples by Lymphoprep density gradient centrifugation. Isolated mononuclear cells were counted on a Countess II cell counter (Thermo Fisher Scientific), lysed in RLT buffer (Qiagen, 600 µl per 1E7 cells) with ß-mercaptoethanol, and stored at − 80 ℃ until further analysis.

### RNA isolation

RNA was extracted from bone marrow lysates (600 µl, corresponding to 1 × 10^7^ cells) with the AllPrep DNA/RNA/Protein Mini kit (Qiagen), according to the protocol provided by the manufacturer. Extracted RNA was eluted in 40 µl of RNAse-free water. The yield and purity were assessed with ultraviolet spectrophotometry, on a Nanodrop spectrophotometer.

### Marker selection

Biomarkers for detecting DTCs were selected, based on findings in previous studies. These markers included mRNAs that encoded four keratins *KRT7, KRT8, KRT18*, and *KRT19*; carcinoembryonic antigen cell adhesion molecule-5 (*CEACAM5*), epithelial cellular adhesion molecule (*EPCAM*); the zinc finger and homeodomain transcription factor *ZEB1*; and the snail family transcriptional repressor 2 (*SNAI2*). We also performed bioinformatic analyses of publicly available mRNA expression data to identify any new markers. Briefly, we downloaded RNA expression data that was publicly available in the Human Protein Atlas [14, 15] in January 2021. We then filtered those data to select mRNAs that were expressed at high levels in normal pancreas and PDACs, but at very low levels in normal bone marrow and peripheral blood mononuclear cells. Candidate mRNAs (*SPINK1, PRSS2, REG1A, MUC1, AGR2, TM4SF1*) were validated experimentally, in a pilot study, in bone marrow samples from both healthy controls and patients. Only the mRNA that encoded serine peptidase inhibitor kazal type 1 (*SPINK1*) passed our evaluation criteria. The final selection of DTC markers is shown in Table 2.

**Table 2:** TaqMan gene expression assays used

**Gene symbol**	**Full gene name**	**TaqMan Assay ID**
*BCR*	Breakpoint cluster region	Hs01036532_m1
*CEACAM5*	Carcinoembryonic antigen-related cell adhesion molecule 5	Hs00944025_m1
*EPCAM*	Epithelial cell adhesion molecular	Hs00158980_m1
*KRT7*	Keratin 7	Hs00559840_m1
*KRT8*	Keratin 8	Hs01595539_g1
*KRT18*	Keratin 18	Hs02827483_g1
*KRT19*	Keratin 19	AI70M8O (custom)
*SNAI2*	Snail family transcription repressor	Hs00950344_m1
*SPINK1*	Serin peptidase inhibitor Kazal type 1	Hs00162154_m1
*ZEB1*	Zinc finger E-box binding homeobox 1	Hs00232783_m1

### Reverse transcription and pre-amplification

Reverse transcription was performed with 1 µg RNA in a total volume of 20 µl with the High-Capacity cDNA Reverse Transcription Kit (Applied Biosystems), according to the manufacturer’s protocol. Pre-amplification was performed to increase the sensitivity for rare mRNAs, in the setting of extensive subsampling. Briefly, cDNA (250 ng in 5 µl) from the transcripts of interest (Table 2) were pre-amplified in a total volume of 50 µl with the TaqMan® PreAmp Master Mix Kit (Applied Biosystems), in 14 amplification cycles, according to the manufacturer’s instructions. The pre-amplified cDNA was diluted to a final volume of 1000 µl.

### Quantitative PCR

**Fig. 1:**
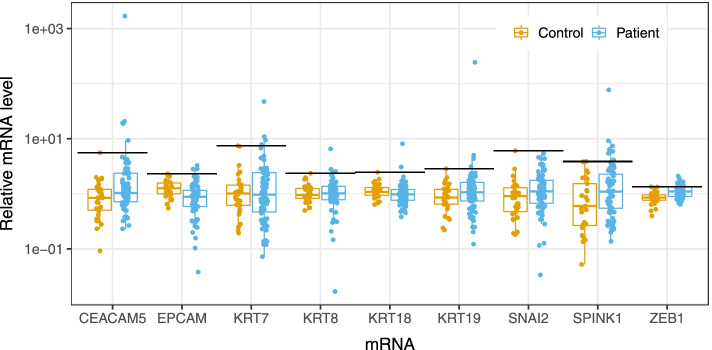
Relative concentrations of DTC markers in bone marrow samples. The relative concentrations of 9 DTC marker mRNAs in bone marrow samples are compared between healthy controls (yellow) and patients with PDAC (blue).The highest level of each mRNA observed in control samples is indicated with a horizontal black line segment. Measurements below detection limits are not shown (5 measurements)

**Fig. 2:**
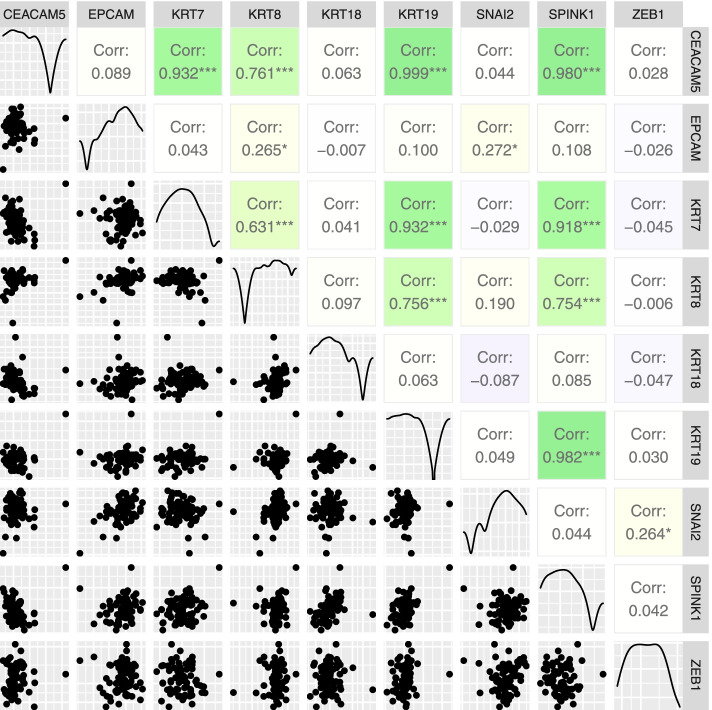
Correlations between relative concentrations of DTC markers. The scatterplots on the left side of the matrix show the median-normalized relative concentrations of DTC markers, plotted on logarithmic axes. Pearson correlation coefficients are shown on the right side of the matrix; the colors and intensities indicate the direction and strengths of the correlations (green: R = 1, white: R = 0, blue: R =  − 1). Statistical significance is indicated with asterisks (***p < 0.001; *p < 0.05). The plots placed on the diagonal show the data densities of the mRNAs indicated in the top bar

Pre-amplified cDNA was quantified in a LightCycler 480 Real-Time PCR System (Roche) with the TaqMan Gene Expression Master Mix (Applied Biosystems) and TaqMan gene expression assays (Applied Biosystems; Table 2), according to the manufacturer’s instructions. The TaqMan assays were all designed with probes or one of the primers spanning exons. Pre-amplified cDNA (5 µl or 6.25 µl; the same volume for whole PCR plates) was placed in a total volume of 25 µl in 96-well plates, and subjected to 40 additional amplification cycles. Cq values were determined with the fit-point method provided in the LightCycler 480 software version 1.5.1. Relative mRNA levels were computed with the 2^ΔΔCq^ method [16], by normalizing against both the *BCR* reference transcript and a calibrator sample included on every plate. A bone marrow sample was considered to contain DTCs (positive DTC status), when the relative concentration of at least one of the mRNA markers was higher than the highest concentration observed for that marker in the healthy control group. To enhance visual comparability, the relative mRNA levels shown in Figs. 1 and 2 were normalized against the median level of each mRNA across all analyzed samples (both controls and patients).

### Statistics

All statistical analyses were performed in Rstudio version 1.4.1103, with R version 4.0.4. Continuous data were compared with the Mann–Whitney test. Associations between categorical data were tested with Fisher’s exact test. Associations between ordered categorical data were examined with the Chi square test for trend.

The date of disease progression was defined as the date that the first radiologic imaging evaluation was scored as “Progressive Disease”, according to the RECIST 1.1 criteria [13]. Univariable survival was assessed with Kaplan–Meier survival estimates, log-rank tests, and univariable Cox regression. Multivariable Cox regression was performed to investigate the independent prognostic value of factors related to overall survival, with backward selection of the variables. Only variables with P-values below 0.1 in the univariable model were included in the initial multivariable model. These variables were: bone marrow DTC status, ECOG performance status (2 vs. 0 or 1), first-line treatment (FOLFIRINOX vs. any other), and clinical T (cT) stage (T4 vs. T1-3). The cT stage was not included in the final model, due to significant associations with two of the other variables. The proportional hazards assumption was checked with the cox.xph function in the survival R package. All tests were two-sided, and *P*-values < 0.05 were considered statistically significant.

This manuscript was prepared according to the REMARK guidelines (recommendations for tumor marker prognostic studies) [17].

## Results

### Disseminated tumor cells in patients with unresectable PDAC

Bone marrow samples (*n* = 73) from 48 patients with locally advanced or metastatic PDAC (Table 1) were examined for the presence of DTCs by measuring the relative expression levels of epithelial and mesenchymal marker mRNAs with RT-qPCR (Fig. 1). We selected the *KRT7, KRT8, KRT18, KRT1*9, *CEACAM5, EPCAM,* and *SPINK1* transcripts as target mRNAs, because they were specifically expressed in epithelial cells. We selected *ZEB1* and *SNAI2* as target mRNAs, because they were mesenchymal-cell specific. We also analyzed bone marrow samples from 30 healthy control individuals to establish thresholds for distinguishing between altered and normal mRNA concentrations in bone marrow (Fig. 1). For each potential DTC marker, we set the highest mRNA level observed in normal bone marrow as the threshold. Based on those thresholds, we observed elevated mRNA levels in zero (*SNAI2*) to 20 (*ZEB1*) bone marrow samples from patients (Table 3). We found that the bone marrow relative levels of *CEACAM5*, *SPINK1*, and *ZEB1* mRNAs were significantly higher in patient samples than in healthy control samples (*p* = 2 × 10^–6^, *p* = 0.03, and *p* = 5 × 10^–5^, respectively; Fig. 1). The levels of some markers (*CEACAM5, KRT7, KRT8, KRT19, SPINK1*) were correlated with each other, although mainly observed in a few samples (Fig. 2). On the other hand, the levels of the mesenchymal markers, *SNAI2* and *ZEB1*, were not correlated with the other markers. Of the 73 bone marrow samples, 39 (53%) had elevated levels of at least one DTC marker.

**Table 3:** Number of BM samples positive for DTC markers

**Marker**	**Positive samples pre-treatment (%)**	**Positive samples in treatment (%)**	**Positive samples in total (%)**
*CEACAM5*	1 (2)	4 (16)	5 (7)
*EPCAM*	2 (4)	1 (4)	3 (4)
*KRT7*	3 (6)	1 (4)	4 (5)
*KRT8*	5 (10)	0 (0)	5 (7)
*KRT18*	2 (4)	0 (0)	2 (3)
*KRT19*	2 (4)	2 (8)	4 (5)
*SNAI2*	0 (0)	0 (0)	0 (0)
*SPINK1*	7 (15)	0 (0)	7 (10)
*ZEB1*	15 (31)	5 (20)	20 (27)
At least one marker	27 (56)	12 (48)	39 (53)
At least two markers	7 (15)	1 (4)	8 (11)
At least three markers	1 (2)	0 (0)	1 (1)
Epithelial multimarker	15 (31)	8 (32)	23 (32)
Total number of samples	48	25	73

Subsequent analyses demonstrated that the epithelial markers were most informative, from a clinical perspective. Thus, we combined the epithelial markers in a multi-marker panel. Among all samples, 23/73 (32%) showed at least one positive result on the panel, including 15/48 (31%) pre-treatment samples and 8/25 (32%) on-treatment samples (Table 3).

Next, we tested for associations between pre-treatment epithelial DTC status and the clinicopathological parameters shown in Table 1. We found that more patients with T2 tumors had DTC-positive samples (*p* = 0.03). Surprisingly, we also observed that more patients with T2 tumors had metastases (M1; *P* = 0.007), compared to patients with larger tumors. Moreover, the cT stage was associated with the type of first-line treatment (fewer patients with small tumors were treated with FOLFIRINOX; *p* = 0.02), but not with the ECOG status.

### Prognostic value of DTCs detected before treatment

**Fig. 3:**
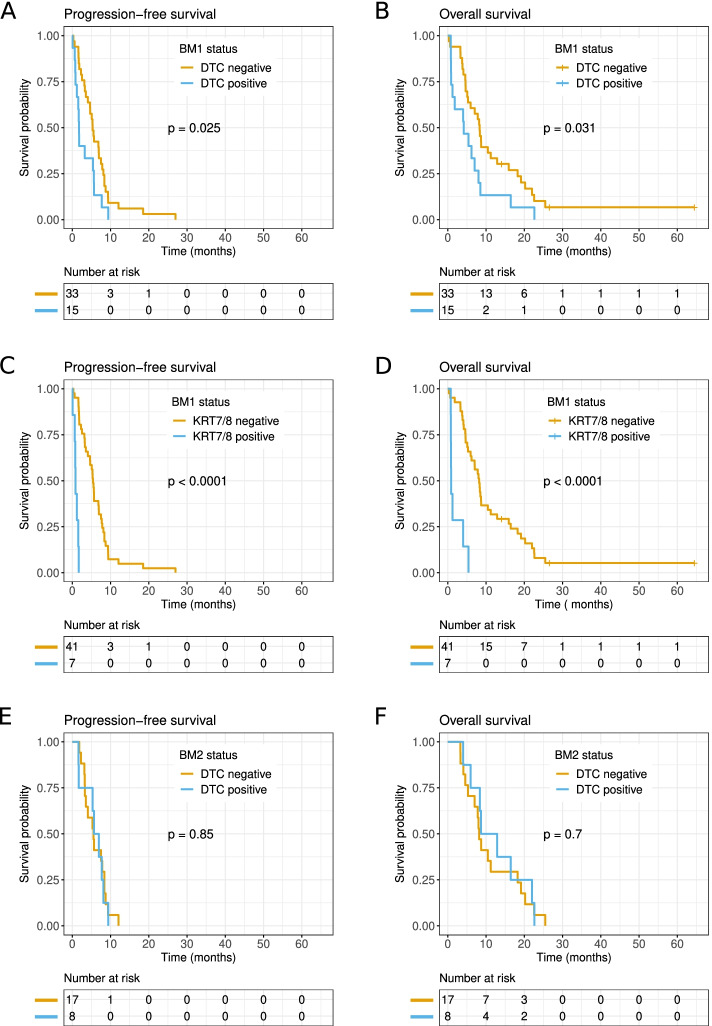
Kaplan–Meier survival estimates stratified by positive/negative DTC detection in bone marrow. **A**, **C**, **E** Progression-free survival and **B**, **D**, **F** overall survival estimates are shown for patients that showed positive (blue) or negative (orange) DTC detection in bone marrow samples acquired **A**-**D** before chemotherapy (BM1) or **E** and **F** during chemotherapy (BM2). DTC detection was based on all epithelial markers (**A**, **B**, **E**, **F**) or restricted to *KRT7* and *KRT8* mRNAs (**C**, **D**). *P*-values were calculated with log-rank tests; the numbers at risk are shown in the panels below each survival curve

Patients were followed for a median of 7.0 months (range 0.30—64 months), which was also the median overall survival time (95% CI: 5.1—8.7 months). The median time to progression was 4.9 months (95% CI: 3.3—5.7 months). Patients with DTCs (based on the epithelial multi-marker assay) before starting chemotherapy had significantly shorter progression-free survival (*p* = 0.03; hazard ratio [HR] = 2.0; 95% CI: 1.1–3.7) and overall survival (*p* = 0.03; HR = 2.0; 95% CI: 1.1–3.8), compared to those without DTCs (Fig. 3A, B; Table 4). When patients were stratified according to single DTC markers, we found that *KRT7* and *KRT8* mRNA contributed most to survival probability (Supplementary Fig. 1). Therefore, we also assessed the progression-free (*p* = 1 × 10^–6^, HR = 22) and overall survival (*p* = 2 × 10^–5^, HR = 7.7) of patients positive for the combination of the *KRT7* and *KRT8* markers before chemotherapy and found their survival significantly shorter than the other patients’ survival (Fig. 3C and D, Table 4). When markers other than *KRT7* and *KRT8* were analyzed individually, none could identify patients with significantly shortened survival (Supplementary Fig. 1).

**Table 4:** Univariable Cox regression overall survival

**Variable**	**Hazard ratio (95% CI)**	***P* value**
Pre-treatment BM DTC status	**2.0 (1.1–3.7)**	**0.034**
Pre-treatment BM KRT7/8 DTC status	**7.7 (3.0–20)**	**0.000024**
Age (> 67 years)	0.92 (0.50–1.7)	0.78
Sex (Female vs male)	0.82 (0.44–1.5)	0.54
Clinical T stage (T4 vs T1/2/3)	**0.49 (0.26–0.92)**	**0.028**
Primary tumor localization (head/body vs tail)	0.46 (0.20–1.1)	0.07
Clinical N stage (N1 vs N0)	0.82 (0.45–1.5)	0.52
Clinical M stage (M1 vs M0)	1.52 (0.75–3.1)	0.25
Metastatic sites (multiple organs vs single)	0.98 (0.46–2.1)	0.95
ECOG status (2 vs 1/0)	**6.4 (2.3–18)**	**0.00031**
First-line treatment (FOLFIRINOX vs other)	**0.47 (0.26–0.87)**	**0.0017**

We performed a univariable Cox regression analysis to assess the prognostic value of pre-treatment bone marrow DTC status and the clinicopathological variables in Table 1. We found that the bone marrow DTC status, cT stage, ECOG status, and the type of first-line treatment were significantly associated with overall survival (Table 4). However, the cT stage was significantly associated with both bone marrow DTC status and the type of first-line treatment; this situation represented a challenge for the multivariable Cox regression. Therefore, we excluded the cT stage from the multivariable analysis. The resulting multivariable model identified three variables with independent prognostic value: pre-treatment DTC status (HR = 2.0), ECOG performance status (HR = 6.7), and the type of first-line therapy (HR = 0.49; Table 5). When the cT stage was included in the model, neither the cT stage nor the DTC status were independent significant predictors of survival. When DTC detection was restricted to only KRT7/8 measurements, the DTC status (HR = 7.9; 95% CI: 3.0–21) and ECOG performance status (HR = 6.5; 95% CI: 2.3–19) were the only independent significant predictors of overall survival.

**Table 5:** Multivariable Cox regression overall survival

**Variable**	**Hazard ratio (95% CI)**	***P* value**
Pre-treatment BM DTC status	**2.0 (1.0–3.8)**	**0.036**
ECOG status (2 vs 1/0)	**6.7 (2.3–19)**	**0.00042**
First-line treatment (FOLFIRINOX vs other)	**0.49 (0.27–0.92)**	**0.027**

### Prognostic value of DTCs detected during treatment

We assessed the prognostic value of DTCs detected after 2 months of chemotherapy. Kaplan–Meier survival estimates revealed no significant differences in progression-free or overall survival, according to in-treatment bone marrow DTC detection (Fig. 3E, F). The same result was obtained when DTC detection was restricted to the *KRT7* and KRT*8* markers; however, only one patient had elevated bone marrow *KRT7* levels and none showed had elevated *KRT8* levels during treatment (Table 3).

## Discussion

This study demonstrated that RT-qPCR detection of DTCs before chemotherapy had prognostic value in patients with inoperable PDAC. The majority of previous studies utilized immunocytochemistry with pan-keratin antibodies to detect DTCs [6]. In studies that included patients with metastatic PDAC, the median rate of DTC-positive findings with immunocytochemistry was 34% (range 14–57) [7, 8, 18], which was quite close to our detection rate of 31% (Table 3) with the epithelial DTC multi-marker assay. In contrast, the only previous study that performed KRT19 RT-PCR for bone marrow DTC detection in PDAC did not identify any patients with DTCs [11]. This apparent discrepancy was probably due to their use of KRT19 mRNA as the only DTC marker; indeed, in the present study, the KRT19 mRNA marker only identified DTCs in 5% of our bone marrow samples (Table 3). Interestingly, KRT19 mRNA was previously used with great success for DTC detection in breast cancer, both in our research group and in other groups [19, 20]. Clearly, the choice and number of markers affect both the DTC detection rate and the prognostic value of the markers. This principle was demonstrated in the present study, when we restricted our analysis to *KRT7* and *KRT8* mRNA (reducing the DTC detection rate to 15%), and in other studies that performed immunocytochemistry with multiple antibodies [21].

The epithelial-to-mesenchymal transition was previously shown to be important for the metastatic process in solid cancers [22, 23]. Accordingly, mesenchymal markers have successfully been used to detect DTCs in breast cancer [24, 25]. However, our current results in PDAC demonstrated that the mesenchymal markers, *SNAI2* and *ZEB1*, were not useful in identifying patients with shortened survival times. In the case of *SNAI2* mRNA, this was due to high background expression levels in normal bone marrow samples. In the case of *ZEB1*, a large number of patient bone marrow samples had elevated mRNA levels (27%; Table 3), regardless of survival. Moreover, the median *ZEB1* level in patient bone marrow samples was significantly higher than the median level observed in normal bone marrow samples (*p* = 5 × 10^–5^). Because the high bone marrow *ZEB1* mRNA levels were not related to clinical outcome, one might speculate whether the high *ZEB1* levels might be caused by factors other than the presence of DTCs.

*KRT7* and *KRT8* mRNA showed much stronger contributions to the prognostic value of DTC detection than the other epithelial markers (Supplemental Fig. 1 and Fig. 3C, D). Interestingly, we noticed that several previous immunocytochemistry studies that used the more narrow-targeted A45-B/B3 antibody (which target KRT7/8/18 [26, 27]) demonstrated that DTC detection had prognostic value in PDAC [7, 18]. In contrast, studies that used the less specific AE3/AE1 antibody cocktail (which target several acidic and basic keratins) failed to show any prognostic value for DTC detection in PDAC [8, 28]. Moreover, in a study on operable breast cancer, a direct comparison of these antibody cocktails demonstrated that DTC detection with the A45-B/B3 antibody had greater prognostic value than DTC detection with the AE3/AE1 antibody [27]. Thus, the choice of DTC markers appeared to affect the ability to identify patients with a poor prognosis. One explanation for this finding could be that different subgroups of DTCs with different keratin expression profiles might reflect different degrees of disease aggressiveness. Another explanation might be that some keratins might be expressed at higher levels in some healthy bone marrow cells, which would increase the rate of false-positive DTC detection [8]. Thus, future experiments are required to clarify the value of different markers for DTC detection, to compare different detection technologies, and to define optimal standardized protocols for DTC detection in PDAC.

To our knowledge, this study was the first to examine DTCs both before and during chemotherapy in patients with PDAC. The proportion of DTC-positive bone marrow samples taken after 2 months of chemotherapy was similar to the corresponding proportion found in the pre-treatment samples (31% versus 32%; Table 3). However, the markers that contributed to DTC detection were quite different before and after treatment commenced. For example, only a single in-treatment sample (4%) showed elevated *KRT7* or *KRT8* concentration. Our findings suggested that KRT7 and KRT8 were the primary markers for identifying true DTCs or DTCs that reflected disease aggressiveness; thus, the strong reduction in KRT7/KRT8-positive samples during therapy may be related to treatment effects. This putative treatment effect might explain why DTC detection with all the epithelial markers lacked prognostic significance during chemotherapy. On the other hand, several previous studies have demonstrated that similar proportions of bone marrow samples were DTC-positive before and after chemotherapy, in both breast and ovarian cancers. Those findings suggested that chemotherapy did not effectively eradicate DTCs in those cancers [29–31]. Another hypothesis is that DTC dormancy may explain DTC persistence, chemoresistance, and late recurrences in breast cancer and other cancers (reviewed in [32, 33]). In PDAC, DTC dormancy might explain why DTCs could be observed in bone marrow samples, despite the rarity of bone metastases. Potentially, bone marrow is not a good “soil” for fostering PDAC cells, but dormancy mechanisms might enable survival in a suboptimal environment. Although DTCs, per se, might not form lethal metastases in PDAC, their presence in bone marrow appears to be associated with increased metastatic capacity in some patients, either due to direct tumor-cell seeding from the bone marrow [34] or because DTC survival in bone marrow is associated with increased metastatic capacity, in general.

Surprisingly, we observed a positive association between DTC detection and small primary tumors (T2) in our study cohort (*P* = 0.03). Moreover, patients with small tumors displayed more metastatic disease (stage M1) and shorter overall survival, compared to patients with larger tumors (*P* = 0.007; Table 4). Based on these observations, one might speculate that our DTC analysis identified a subgroup of patients with small, but particularly aggressive, primary tumors. Despite the established relationships between cT stage, M stage, and survival [35], other researchers have identified similar subgroups of patients with PDAC that have small, but aggressive, node-positive tumors [36]. However, due to the small size of our study cohort, these unexpected observations should be interpreted with caution.

Clinically, DTC detection might not be highly useful in metastatic PDAC, due to the extremely poor prognosis and limited treatment options for this patient group. However, DTCs have also been shown to provide prognostic information in the non-metastatic setting. In that setting, DTC detection might represent an approach for selecting patients that might benefit from adjuvant or neoadjuvant chemotherapy [7]. Clinical intervention studies are required to establish appropriate applications for DTC detection in the future.

## Conclusions

This study showed that the detection of DTCs before initiating chemotherapy, but not during chemotherapy, could provide prognostic value in patients with unresectable PDAC. We found that the DTC markers *KRT7* and *KRT8* provided stronger prognostic information than other epithelial markers. In contrast, the investigated mesenchymal markers did not contribute to prognostic value. Further research is required to clarify the roles of different DTC markers, to optimize and standardize DTC detection methods, and to establish the clinical utility of DTC detection in diagnostics.

## Supplementary Information

**Additional file 1: Supplementary Figure 1.** Kaplan-Meier overall survival estimates stratified according to single DTC markers.

## Data Availability

The datasets generated and analysed during the current study are not publicly available due to their sensitive nature (potentially identifying and sensitive personal information) and related limitations in the written informed consent (not mentioning public availability of collected data), but are available from the corresponding author on reasonable request.
