# Supplementary material for: Prognostic value of disseminated tumor cells in unresectable pancreatic ductal adenocarcinoma: a prospective observational study
Source: BMC Cancer. 2022 Jun 3;22:609. doi: 10.1186/s12885-022-09714-x (PMC9166481; doi:10.1186/s12885-022-09714-x)
Supplement: Supplementary file 1 — Additional file 1: Supplementary Figure 1. Kaplan-Meier overall survival estimates stratified according to single DTC markers. [file 12885_2022_9714_MOESM1_ESM.pdf]

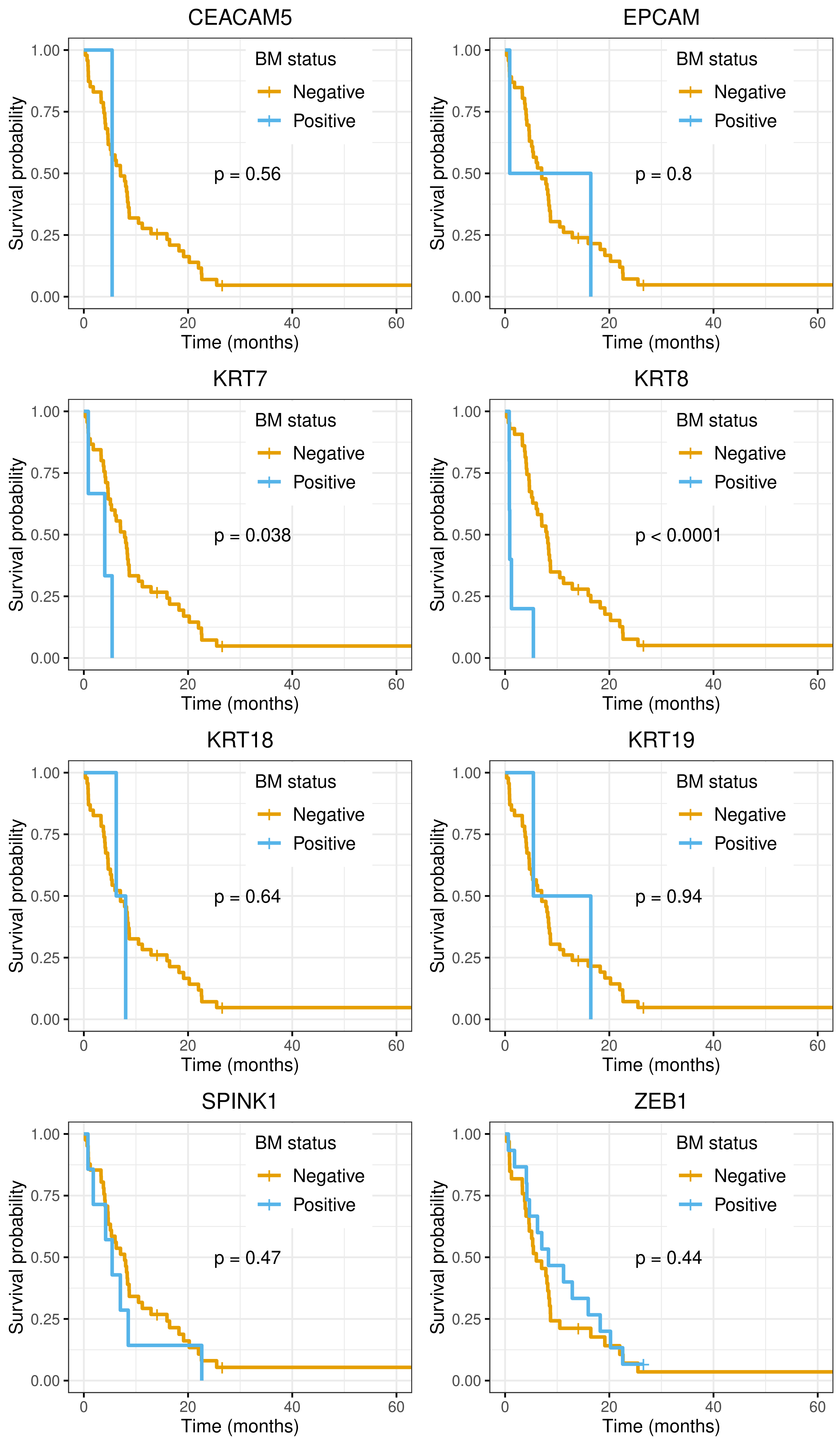

Supplementary Figure 1: Kaplan-Meier overall survival estimates stratified according to single DTC markers.
